# Preservation Analysis of Macrophage Gene Coexpression Between Human and Mouse Identifies PARK2 as a Genetically Controlled Master Regulator of Oxidative Phosphorylation in Humans

**DOI:** 10.1534/g3.116.033894

**Published:** 2016-08-24

**Authors:** Veronica Codoni, Yuna Blum, Mete Civelek, Carole Proust, Oscar Franzén, Cardiogenics Consortium, IDEM Leducq Consortium CADGenomics, Johan L. M. Björkegren, Wilfried Le Goff, Francois Cambien, Aldons J. Lusis, David-Alexandre Trégouët

**Affiliations:** * Sorbonne Universités, Université Pierre et Marie Curie, Paris 06, Institut National de la Santé et de la Recherche Médicale, Unité Mixte de Recherche en Santé 1166, F-75013 Paris, France; † Institute for Cardiometabolism and Nutrition, F-75013 Paris, France; ‡ Division of Cardiology, Department of Medicine, David Geffen School of Medicine, University of California, Los Angeles, California 90095; § Department of Biomedical Engineering, Center for Public Health Genomics, University of Virginia, Charlottesville, Virginia 22908; ** Department of Genetics and Genomic Sciences, Icahn School of Medicine at Mount Sinai, New York, New York 10029; †† Icahn Institute of Genomics and Multiscale Biology, Icahn School of Medicine at Mount Sinai, New York, New York 10029; ‡‡ Department of Medical Biochemistry and Biophysics, Karolinska Institutet, Stockholm 141 52, Sweden

**Keywords:** gene expression network analysis, eQTL analysis, macrophages, cross-species comparison, *trans* genetic effects

## Abstract

Macrophages are key players involved in numerous pathophysiological pathways and an in-depth characterization of their gene regulatory networks can help in better understanding how their dysfunction may impact on human diseases. We here conducted a cross-species network analysis of macrophage gene expression data between human and mouse to identify conserved networks across both species, and assessed whether such networks could reveal new disease-associated regulatory mechanisms. From a sample of 684 individuals processed for genome-wide macrophage gene expression profiling, we identified 27 groups of coexpressed genes (modules). Six modules were found preserved (*P* < 10^−4^) in macrophages from 86 mice of the Hybrid Mouse Diversity Panel. One of these modules was significantly [false discovery rate (FDR) = 8.9 × 10^−11^] enriched for genes belonging to the oxidative phosphorylation (OXPHOS) pathway. This pathway was also found significantly (FDR < 10^−4^) enriched in susceptibility genes for Alzheimer, Parkinson, and Huntington diseases. We further conducted an expression quantitative trait loci analysis to identify SNP that could regulate macrophage OXPHOS gene expression in humans. This analysis identified the PARK2 rs192804963 as a *trans*-acting variant influencing (minimal *P*-value = 4.3 × 10^−8^) the expression of most OXPHOS genes in humans. Further experimental work demonstrated that PARK2 knockdown expression was associated with increased OXPHOS gene expression in THP1 human macrophages. This work provided strong new evidence that *PARK2* participates to the regulatory networks associated with oxidative phosphorylation and suggested that *PARK2* genetic variations could act as a *trans* regulator of OXPHOS gene macrophage expression in humans.

Macrophages play critical roles in several human physiological processes, including atherosclerosis (Madamanchi *et al.* 2005), inflammation (Akira *et al.* 2013), insulin resistance (Jacobi *et al.* 2012), oxidative phosphorylation (Tavakoli *et al.* 2013), and pathogen clearance (Murray and Wynn 2011). As a consequence, their uncontrolled dysfunction has been associated with various human diseases, such as autoimmune disorders (Casanova and Abel 2009; Nathan and Ding 2010), Alzheimer disease (Saresella *et al.* 2014), coronary artery disease (Ghattas *et al.* 2013), obesity (Jacobi *et al.* 2012), and type 2 diabetes (Eguchi and Manabe 2013; Van Gassen *et al.* 2015). Despite intensive research, the mechanisms of macrophage activation and regulation, and their impact on disease susceptibility, are not fully understood; a prerequisite for devising efficient therapeutic strategies oriented toward the aforementioned diseases. A possible strategy to uncover novel pathophysiological roles for genes within specific cell types is to assess the impact of genetic variations on transcript abundance (*i.e.*, gene expression) and map the results to disease-associated loci (Chen *et al.* 2008; Fairfax and Knight 2014). In addition, gene expression network and gene annotation enrichment analyses may identify highly coregulated genes and reveal new partners of physiopathological interest (Subramanian *et al.* 2005; Schadt 2009; Rotival *et al.* 2011). This approach may be conducted across different species to achieve deeper understanding of regulatory mechanisms and reveal novel gene functions (Oldham *et al.* 2006; Miller *et al.* 2010; Hansen *et al.* 2014) and may be integrated within efficient multilayers or a systems biology approach (Bunyavanich and Schadt 2015).

Here we used a system biology approach to better understand regulatory mechanisms in human and mouse macrophages. To reduce the risk of focusing on spurious or irrelevant networks, we checked the networks (or modules) identified in human macrophages in mouse macrophages. The rationale of this approach was that gene coexpression networks that are conserved across both species are more likely to reflect key biological functions (Hansen *et al.* 2014). Gene annotation enrichment analysis was then performed on the identified modules to assess whether they correspond to physiopathologically relevant functions or pathways. Finally, using genome-wide single nucleotide polymorphism (SNP) data, we identified genetic variants influencing gene expression within conserved modules. Our specific aim was to identify *trans*-acting SNPs that affect the transcriptome of conserved modules, as these variants may reveal the existence of master regulator genes with pleiotropic effects.

## Materials and Methods

This work relied on two genome-wide macrophage expression resources, one performed on human samples from the Cardiogenics Transcriptome Study (CTS) and the second on mice from the Hybrid Mouse Diversity Panel. The methodologies used for obtaining and processing CTS data have been previously described in detail (Rotival *et al.* 2011; Charchar *et al.* 2012; Garnier *et al.* 2013). The present work was based on the analysis of 684 individuals with macrophage gene expression. Mice expression data were obtained from 86 mice, of which the extraction and preprocessing analyses have been extensively described in Orozco *et al.* (2012).

### Macrophages isolation and RNA extraction (human)

Macrophages were derived -monocytes. Monocytes were isolated from whole blood positive selection with CD14 microbeads (Miltenyi) according to the manufacturer’s instructions. Monocyte purity was measured as the percentage of CD14+ cells analyzed by flow cytometry. Macrophages were obtained from culturing of monocytes for 7 d in macrophage-SFM medium (Gibco/Invitrogen) with 50 ng/ml^−1^ recombinant human M-CSF (R&D Systems). RNA was extracted from both monocytes and macrophages with TRIzol, followed by clean-up with RNeasy columns (Qiagen, Venlo, The Netherlands) and DNase-based treatment (Charchar *et al.* 2012).

### Human expression data

Gene expression profiling was performed using Illumina’s Human Ref-8 Sentrix Bead Chip arrays (Illumina, San Diego, CA) containing 24,516 probes corresponding to 18,311 distinct genes and 21,793 RefSeq annotated transcripts. mRNA was amplified and labeled using the Illumina Total Prep RNA Amplification Kit (Ambion, Austin, TX). After hybridization, array images were scanned using the Illumina BeadArray Reader, and probe intensities were extracted using the Gene expression module (version 3.3.8) of Illumina’s Bead Studio software (version 3.1.30). Expression signals were background corrected using GenomeStudio software. Probes were included in the analysis if their expression was considered detected (Illumina detection *P* < 0.01) in at least 90% of samples. After removing nonwell-characterized probes, a total of 15,539 probes corresponding to 12,502 distinct genes remained for the analysis. Variance stabilization transformation was applied to the raw intensities and quantile normalization was done in the R statistical environment with the Lumi package (Lin *et al.* 2008; Du *et al.* 2008). Principal variance components analysis was used to identify main factors contributing to the variability of expression data. Given the strong influence of the variables center, sample batches, date of hybridization, and microarray, we performed an adjustment on these factors using the function Combat implemented in the sva R package (Leek *et al.* 2012).

### Human genotype data

CTS participants were typed for genome-wide genotype data using the Human Custom 1.2 M and the Human 610 Quad Custom arrays from Illumina. SNPs with genotyping call rate < 99%, minor allele frequency (MAF) < 0.01, or showing significant (*P* < 10^−5^) deviation from Hardy–Weinberg equilibrium were filtered out. This led to 506,290 quality control (QC)–validated autosomal SNPs. Individuals were excluded according to the following criteria: genotyping rate < 95% close relatedness as suspected from pairwise clustering of identity by state distances and multidimensional scaling implemented in PLINK (Purcell *et al.* 2007), and genetic outliers of non-European ancestry detected by principal components analysis as implemented in the EIGENSTRAT program (Price *et al.* 2006). The 506,290 QC-checked SNPs were then used for imputing 11,672,179 autosomal SNPs from the 1000 Genomes February 2012 release reference dataset. For this, MACH (version 1.0.18.c) software was used (Li *et al.* 2010). All SNPs with acceptable imputation quality *r*^2^ > 0.3 (Johnson *et al.* 2013) and imputed MAF > 0.01 were kept for genotype-expression association analysis (*N* = 8,989,527).

### Macrophage mouse expression study

Macrophages were primary intraperitoneal macrophages in control conditions, isolated and processed as in Orozco *et al.* (2012).

Total RNA extracted from 86 strains was profiled with Affymetrix Mouse Genome HT MG-430A arrays. The image data were processed using the Robust Multichip Average method to determine the hybridization signal for each gene. A total of 17,962 probes corresponding to 12,242 genes were available for further analysis.

### Mouse genotyping

Mouse inbred strains were genotyped using the Mouse Diversity Array, which contains probes for 623,124 SNPs (Yang *et al.* 2009). After filtering the SNPs for MAF < 5% and genotype missingness rate < 5%, 205,539 SNPs remained for association testing.

### Data combination

Human and mouse macrophage gene expressions have been preprocessed separately, as described above. The probe-level measurements were converted into gene-level measurements in both datasets to allow comparison across different platforms. The probe within a gene that had the maximum average expression across samples was used to represent that gene. In order to compare gene expressions between the different species, the ENSEMBL Gene ID was used to derive mouse orthologous to human genes. The result of this step was an overall of 7890 genes commonly expressed in human and in mouse gene expression datasets.

Human and mouse macrophage samples were clustered separately, based on their Euclidian distance to detect outlier observations. A total of 19 human and 7 mouse samples were removed as outliers for further analysis.

From the 665 (= 684 − 19) CTS individuals analyzed for their expression data, 576 individuals also had QC genome-wide genotype data.

### Gene coexpression network construction for human macrophages

A weighted gene coexpression network analysis (WGCNA) was conducted on the human macrophage expression dataset, composed of 7890 genes and 665 samples, to identify modules of coexpressed genes. To construct the network, the absolute values of correlation coefficients [biweight midcorrelation Zheng *et al.* (2014)] were calculated for all possible gene pairs. Values were entered into a matrix, and the data were weighted into an adjacency matrix such that it followed an approximate scale-free topology (estimated β power = 5). Finally, the topological overlap matrix (TOM) was converted from the adjacency matrix and used to derive a TOM-based distance matrix for the next hierarchical clustering of expressions. We performed an average hierarchical clustering with the TOM-based metric as distance and identified groups of highly correlated human genes cutting the branches of dendrogram by dynamic tree cut algorithm (Langfelder *et al.* 2008), which iteratively searches for stable branch size and selects cluster based on the shape of each branch. We set up deepSplit = 3, min ModuleSize = 50 as parameters for the dynamic tree cut function (others were default values).

The expression of each identified human module was then summarized in terms of their Module Eigengene (ME) value, calculated as the first principal component derived from all gene expression belonging to the given module. To assess the coexpression similarity between identified modules, a hierarchical clustering was performed on module eigengene expressions. At a height cut-off of 0.15, corresponding to a pairwise correlation of 0.85, no strong similarity was observed between modules.

We also quantified the contribution of a gene to a module by the module membership metric, defined as the correlation between a single gene’s expression and the specific module eigengene ME, referred to as the kME value thereafter.

### Preservation analysis on mouse macrophages dataset

In order to assess how well a human module was preserved in mouse macrophage data, we used mouse expression data to compute the ME and kME metrics derived from the human modules genes’ composition. Preservation of human modules in mouse data was then determined using both human and mouse kME values. “Consistent genes” between species were then defined as the set of genes in each human module that had concordant sign of kME values in both datasets. Then, the percentage of consistent genes between species was computed for each human module; the higher the percentage, the more preserved the modules.

A permutation procedure was used to assign a *P*-value to this measure of preservation between the two datasets. The null hypothesis was that the proportion of consistent genes observed for each human module was no better than the corresponding proportion of consistent genes of modules derived from random clustering. To evaluate this hypothesis, human gene identifiers were randomly permuted so that human gene modules of the same size but with random gene composition were generated. A total of 10,000 such bootstrap iterations were performed and the percentage of consistent genes of each human random module assignment between the two species was calculated for each iteration. The probability of the null hypothesis was then calculated as the proportion of bootstrap iterations in which the percentage of consistent genes of random modules across species was greater than that of the human observed ones. We also evaluated module preservation using alternative, more complex methods based on composite statistics, *Z*-summary, and median rank statistics, derived from the density and connectivity of the modules, as implemented in the WGCNA R package (Langfelder *et al.* 2011). These statistics summarize the evidence that a human module is preserved more significantly than a random sample of genes.

### Gene ontology and pathway enrichment analysis

To study the biological relevance of consistent genes, we performed a functional enrichment analysis using the Database for Annotation, Visualization and Integrated Discovery tool (DAVID; Huang *et al.* 2009), and the human gene annotation list as background. GO, KEGG, REACTOME, and PANTHER databases were interrogated among the consistent genes of preserved modules.

### 1000 Genomes imputation-based expression quantitative trait loci analysis in human macrophages

Associations between imputed genotypes and expression were computed using a linear regression model where the imputed allele dosage was used as covariate to assess SNP effect. Analyses were conducted by use of the MatrixEQTL R package (Shabalin 2012), adjusting for sex, age, and potential contaminations cell types (*i.e.*, CD4+, CD8+, CD19+, CD56+, CD66b+, erythroblasts, and megakaryocytes counts). Expression quantitative trait loci (eQTL) effects were considered as *cis* if the SNP was located within a 10^6^ bp distance upstream or downstream from probe sequence. Otherwise, they were considered as *trans*. A statistical threshold of 5 × 10^−8^ was used to declare significance. A total of 576 individuals with both imputed genotypes and macrophage gene expressions were available for eQTL analysis.

### eQTL analysis in mouse macrophages

eQTL mapping was performed using FaST-LMM (Lippert *et al.* 2011), a linear mixed-model method that is able to account for the uncontrolled population structure of the data.

### RNA interference-mediated PARK2 silencing using small interference RNA

Human THP-1 monocytic cells (from the American Type Culture Collection) were cultured and differentiated into macrophage-like cells as previously described (Larrede *et al.* 2009). PARK2 knockdown (KD) THP-1 macrophages were obtained by application of small interference RNA (siRNA) oligonucleotides (Eurogentec) targeted to the complementary DNA sequence of the human *PARK2* gene (Genebank: NM_004562). The sequences of the siRNA were 5′-UUGCUUAGACUGUUUCCACUUAUAC-UU-3′ and 5′-P-GUAUAAGUGGAAACAGUCUAAGCAA-UU-3′, respectively.

### RNA extraction and gene expression analysis

Forty-eight hours following transfection with siRNA, control and PARK2 KD cells were washed twice with cold PBS and total RNA was extracted using an RNeasy mini kit (Qiagen) according to the manufacturer’s instructions. Reverse transcription of RNA and real-time quantitative PCR using a LightCycler LC480 (Roche) were performed as previously described (Larrede *et al.* 2009). Primers used for quantification of *PARK2*, *COX6A*, and *COX6C* mRNA are indicated in Supplemental Material, Table S1. Expression data were based on the crossing points calculated with the software for LightCycler data analysis and corrected for PCR efficiencies of the target and the reference gene. mRNA levels were normalized to housekeeping genes (δ-aminolevulinate synthase, hypoxanthine phosphoribosyltransferase, and α-tubulin). Data were expressed as a fold change in mRNA expression relative to control values. Four independent experiments were conducted in triplicates. Nonparametric Mann–Whitney test was used to test for the impact of PARK2 KD on gene expressions.

### Ethic approval and consent to participate

The CTS was approved by the Institutional Ethical Committee of each Cardiogenics participating center. All individuals gave written informed consent. All animal work was conducted according to relevant national and international guidelines and was approved by the UCLA Animal Research Committee, the UCLA IACUC.

### Data availability

Mouse macrophage data used in this study are deposited in the NCBI GEO repository (http://www.ncbi.nlm.nih.gov/geo/) under the accession number GSE38705. Cardiogenics macrophage expression data are deposited in the European Genome-phenome Archive (https://www.ebi.ac.uk/ega/) under the accession number EGAS00001000411.

## Results

**Figure 1:**
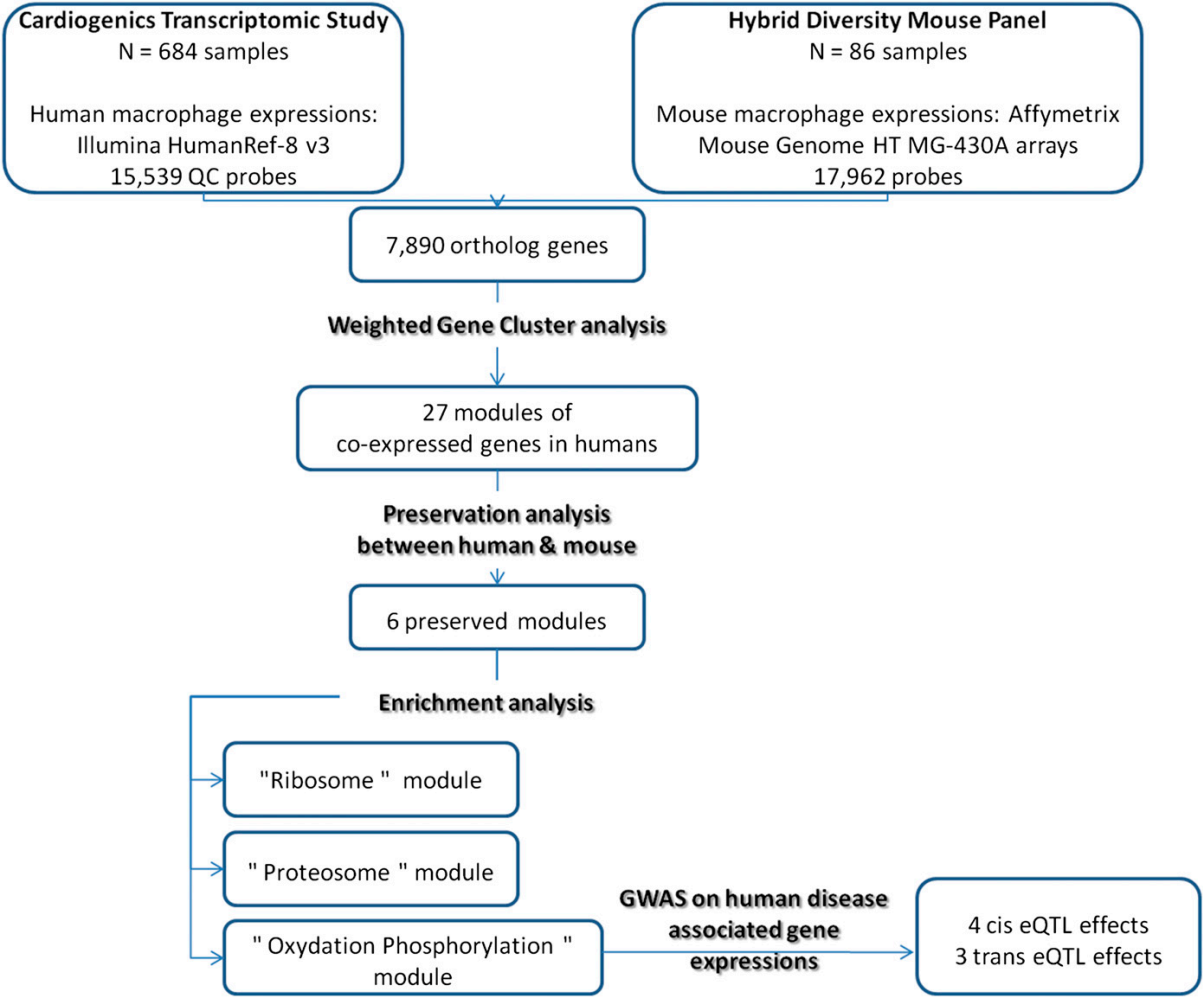
Analysis workflow of the present study.

We identified 7890 genes that are orthologous in humans and mice and for which we had expression data in both species; these genes serve as the basis of our analysis. The overall analysis workflow adopted in this work is summarized in Figure 1.

### Gene expression modules in human macrophage

Human gene expression data were first investigated using WGCNA (Langfelder and Horvath 2008) to identify modules (or clusters) of genes whose expressions were highly correlated (see *Materials and Methods*). Twenty seven modules encompassing 6802 genes (86%) were identified (Table 1). The remaining 1088 genes were only weakly correlated with other genes and were not included in any module. The size of the identified modules, labeled with numbers to allow their distinction, ranged from 62 (M27) to 967 (M1) (Table 1). Each module was then characterized by its first principal component (ME) (Langfelder *et al.* 2011) computed from the covariance matrix of expression levels of the genes in the module. The percentage of module expression variability explained by MEs ranged from 17% (M3 module of size 449) to 30% (M26 module of size 67) (Table 1). The contribution of a given gene to its module was defined as the correlation of its expression with that of its module-associated ME (the kME metric; Langfelder *et al.* 2011).

**Table 1:** Characteristics of the 27 modules identified in human macrophage data

Modules	Size	ME %	% Consistent Genes
M27	62	23.4	0.71
M26	67	30.1	0.64
M25	68	28.1	0.49
M24	73	26.5	0.82
M23	90	28.1	0.80
M22	91	25.8	0.79
M21	96	26.2	0.82
M20	98	28.7	0.63
M19	131	28.4	0.93
M18	141	20.7	0.42
M17	158	24.1	0.82
M16	159	25.2	0.53
M15	160	24.7	0.86
M14	185	21.4	0.67
M13	186	25.8	0.51
M12	209	29.3	0.45
M11	217	27.1	0.60
M10	270	28.6	0.60
M9	282	24.4	0.70
M8	292	25.0	0.58
M7	295	22.9	0.60
M6	369	22.0	0.65
M5	394	26.8	0.69
M4	439	22.6	0.46
M3	449	17.1	0.54
M2	854	18.1	0.61
M1	967	24.2	0.74
M0 (unassigned genes)	1088	3.2	0.70

Size is the number of genes composing the module. ME % is the percentage of gene expression variability explained by the module eigengene (ME). The last “module” in this table corresponds to isolated genes (*i.e.*, genes not assigned to any modules).

### Preservation analysis of human modules in mouse

**Figure 2:**
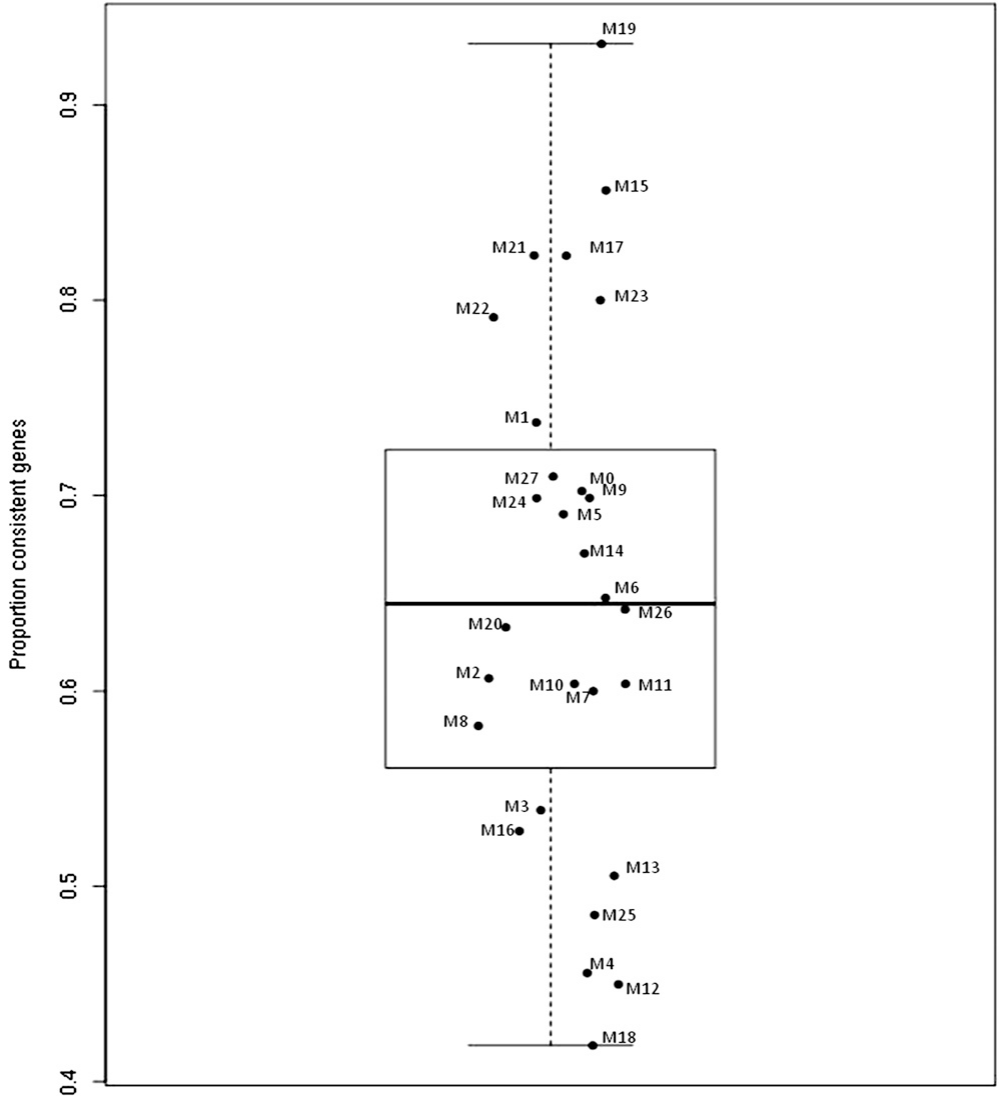
Distribution of the percentage of consistent genes across identified human macrophage gene expression modules.

In a second step, we assessed whether the 27 modules identified in human macrophages were preserved in the mouse model. For this, we first partitioned the mouse genes into the same module assignments as in humans. We then computed mouse-specific MEs and kMEs. For each module, we then calculated the percentage of genes exhibiting similar kME between human and mouse (see Figure S1 for illustrative examples). Consistent genes are those with similar kME sign across the two species. A higher percentage of consistent genes indicates the preservation of human module in the mouse dataset. Figure 2 shows the distribution of the percentage of consistent genes for each human modules. This percentage ranged from 42% for the M18 module to 93% for the M19 module, with a mean of ∼65%. From this distribution, six modules (M19, M15, M21, M17, M23, and M22) were considered preserved between human and mouse. We also used additional metrics based on other network properties to assess module preservation, such as the composite *Z*-summary and *Z* median rank statistics (Langfelder *et al.* 2011). Their application led to similar results with consistent identification of the same preserved modules between humans and mice (Figure S2). We also performed bootstrap analyses (10,000 bootstrap samples) to assess the statistical significance of the observed proportion of consistent genes for these six modules (see *Materials and Methods*). For each module, none of the bootstrap samples produced proportions of consistent genes that were higher than the observed ones (*P* < 10^−4^). Gene composition of the six identified preserved modules is shown in Table S2.

### Enrichment analysis of preserved modules

Pathway analysis was then applied to the six most preserved modules to assess whether they were enriched for genes belonging to specific biological pathways. Enrichment analysis was performed using the DAVID software (Huang *et al.* 2009) interrogating the GO, KEGG, REACTOME, and PANTHER databases (see *Materials and Methods*). Results of the enrichment analysis are provided in Table 2. At a false discovery rate (FDR) of 5%, three modules were found to be significantly enriched in genes belonging to specific biological pathways. The M21 module was significantly enriched (FDR = 8.85 × 10^−27^) for genes coding for ribosomal-associated proteins and the M15 module for proteasome-related genes (FDR = 1.37 × 10^−3^). The M15 was also significantly enriched (FDR = 0.003) for genes belonging to the oxidation phosphorylation (OXPHOS) pathway, as was the M19 module (FDR = 3.28 × 10^−11^) but much more significantly. OXPHOS genes present in the M15 and M19 modules, with sizes 10 and 18 respectively, were not overlapping, which is expected given the way modules were constructed.

**Table 2:** Enrichment analysis on the most preserved human modules

Preserved Module	Pathway	Enrichment Score	FDR	No. of Genes	Genes
M19 (*n*_c_ = 131)	Oxidative phosphorylation	8.92	3.28 × 10^−11^	18	NDUFB3, ATP5J2, COX7A2, NDUFB7, NDUFA9, COX8A, ATP5G2, UQCRQ, NDUFA1, COX6C, NDUFB2, NDUFA11, SDHB, COX6B1, COX6A1, ATP5I, COX17, ATP5J
	Huntington disease		8.85 × 10^−8^	17	NDUFB3, CLTA, COX7A2, POLR2L, NDUFB7, NDUFA9, COX8A, POLR2I, ATP5G2, UQCRQ, NDUFA1, COX6C, NDUFB2, SDHB, COX6B1, COX6A1, ATP5J
	Diabetes pathway		3.62 × 10^−7^	20	NDUFB3, ATP5J2, NDUFB11, NDUFB7, NDUFA9, COX8A, NDUFA13, UQCRQ, NDUFA1, NDUFA12, COX6C, NDUFB2, NDUFA11, SDHB, IDH3G, COX6B1, DAD1, COX6A1, ATP5I, ATP5J
	Parkinson disease		1.31 × 10^−6^	14	NDUFB3, COX7A2, NDUFB7, NDUFA9, COX8A, ATP5G2, NDUFA1, UQCRQ, NDUFB2, COX6C, SDHB, COX6B1, COX6A1, ATP5J
	Alzheimer disease		2.57 × 10^−5^	14	NDUFB3, COX7A2, NDUFB7, NDUFA9, COX8A, ATP5G2, NDUFA1, UQCRQ, NDUFB2, COX6C, SDHB, COX6B1, COX6A1, ATP5J
M15 (*n*_c_ = 137)	Oxidative phosphorylation	4.17	0.003	10	NDUFA4, NDUFV2, SDHD, NDUFAB1, ATP5F1, ATP5C1, ATP5L, NDUFC1, ATP5H, PPA2
	Parkinson disease		0.025	9	NDUFA4, CASP9, NDUFV2, SDHD, NDUFAB1, ATP5F1, ATP5C1, NDUFC1, ATP5H
	Huntington disease		0.044	10	NDUFA4, CASP9, NDUFV2, SDHD, NDUFAB1, ATP5F1, ATP5C1, NDUFC1, ATP5H, TBPL1
	Alzheimer disease		0.14	9	NDUFA4, CASP9, NDUFV2, SDHD, NDUFAB1, ATP5F1, ATP5C1, NDUFC1, ATP5H
	Diabetes pathway		0.30	13	NDUFA4, SEC11C, NDUFAB1, ATP5F1, NDUFC1, DLD, SDHD, NDUFV2, ATP5C1, ATP5L, ATP5H, SRP9, FH
	Proteasome	3.72	1.37 × 10^−3^	8	UBE2N, PSMD14, UBE2D2, PSMD12, PSMC2, UCHL5, PSMD6, PSMD7
M21 (*n*_c_ = 96)	Ribosome	26.70	8.85 × 10^−27^	22	RPL18, RPSA, RPL17, RPL35, RPS9, RPL27, RPL38, RPS6, RPS5, RPS25, RPS19, RPL31, RPL22, RPL3, RPL5, RPS10, RPL11, RPL4, RPS20, RPL10A, UBA52, RPS24

Modules *M17*, *M23*, and *M22* were not significantly enriched for any specific biological pathways. FDR, false discovery rate; *n*_c_, number of consistent genes in the module.

As the OXPHOS module was enriched in candidate genes for diabetes and neurological disorders, we decided to further focus on the following section on the genetic components of these genes, with the aim of identifying additional genetic information that could be relevant for these human diseases. Results of the corresponding analyses for the ribosome and proteasome pathways genes are shown in Table S3, Table S4, and Table S5.

### Genetic regulation of the OXPHOS genes

We further investigated whether the human macrophage expression of the 28 OXPHOS genes from the M19 and M15 modules could be under genetic control. A genome-wide association study (GWAS) analysis was conducted on the human macrophage expression of each of the 28 OXPHOS genes in the CTS samples. Significant *cis* eQTLs were detected for four genes, *COX6B1*, *COX8A*, *NDUFB7*, and *NDUFC1*, with minimum association *P*-values of *P* = 2.1 × 10^−9^, *P* = 1.9 × 10^−8^, *P* = 6.8 × 10^−9^, and *P* = 4.3 10^−9^, respectively (Table S6). Three *trans*-associations were also detected:

*LPCAT1*: The minor T allele of rs115960372 SNP at the *LPCAT1* gene on chromosome 5 was associated with increased *NDUFV2* gene expression (*P* = 1.9 × 10^−9^) (Table 3). It also showed suggestive evidence of association with increased expression of two other OXPHOS genes, *ATP5C1* (*P* = 5.64 × 10^−5^) and *NDUFAB1* (*P* = 2.12 × 10^−6^) (Table 3). None of the other studied genes were associated with the *LPCAT1* rs115960372.

**Table 3:** Association of *LPCAT1* rs115960372 with human macrophage expression of 28 OXPHOS genes

Gene	Probes	Chr	Probe_Start	Probe_End	β*^a^*	SEM	*P* Value
M19 OXPHOS genes							
SDHB	*ILMN_1667257*	1	17,476,541	17,476,590	0.024	0.0135	0.080
NDUFB3	*ILMN_2119945*	2	201,943,702	201,944,702	0.045	0.0168	6.82 × 10^−3^
COX17	*ILMN_2187718*	3	119,396,160	119,396,209	0.000	0.0195	0.982
ATP5I	*ILMN_1772506*	4	678,058	678,107	0.0144	0.0125	0.250
UQCRQ	*ILMN_1666471*	5	132,174,747	132,174,796	0.013	0.0152	0.389
COX7A2	*ILMN_1701293*	6	75,950,943	75,951,943	0.019	0.0145	0.182
ATP5J2	*ILMN_2307883*	7	99,217,929	99,217,978	0.028	0.0156	0.075
NDUFB2	*ILMN_2117330*	7	140,402,713	140,402,762	−0.008	0.0172	0.635
COX6C	*ILMN_1654151*	8	100,904,152	100,904,201	0.006	0.0116	0.611
COX8A	*ILMN_1809495*	11	63,742,263	63,743,263	0.020	0.0152	0.183
NDUFA9	*ILMN_1760741*	12	4,796,151	4,796,200	0.040	0.0175	0.021
ATP5G2	*ILMN_1660577*	12	54,063,071	54,063,120	−0.027	0.0185	0.137
COX6A1	*ILMN_1783636*	12	120,876,242	120,876,291	0.024	0.0164	0.146
NDUFA11	*ILMN_2175712*	19	5,945,952	5,946,001	−0.018	0.0162	0.259
NDUFB7	*ILMN_1813604*	19	14,816,068	14,817,068	−0.002	0.0159	0.876
COX6B1	*ILMN_2154671*	19	36,139,232	36,139,281	−0.019	0.0132	0.150
ATP5J	*ILMN_2348093*	21	28,180,168	28,180,217	−0.010	0.0154	0.509
NDUFA1	*ILMN_1784286*	X	119,005,887	119,005,936	0.017	0.0139	0.232
M15 OXPHOS genes							
ATP5F1	*ILMN_1721989*	1	112,003,559	112,003,608	0.033	0.0130	0.0110
PPA2	*ILMN_1687785*	4	106,292,029	106,293,029	0.046	0.0205	0.0256
NDUFC1	*ILMN_1733603*	4	140,216,254	140,217,254	0.050	0.0175	4.70 × 10^−3^
NDUFA4	*ILMN_1751258*	7	11,006,668	11,006,717	0.033	0.0171	0.0522
ATP5C1	*ILMN_1701269*	10	7,801,069	7,801,118	0.074	0.0183	5.64 × 10^−5^
SDHD	*ILMN_1698487*	11	111,966,144	111,966,193	0.049	0.0201	0.015
ATP5L	*ILMN_2079285*	11	118,280,301	118,280,350	0.049	0.022	0.029
NDUFAB1	*ILMN_2179018*	16	23,684,934	23,684,983	0.091	0.0190	2.12 × 10^−6^
ATP5H	*ILMN_1666372*	17	75,524,607	75,524,656	0.029	0.0136	0.031
NDUFV2	*ILMN_2086417*	18	9,126,871	9,127,871	0.104	0.0170	1.89 × 10^−9^

a Effect of the minor rs115960372 T allele on gene expression. Its allele frequency was 0.10 and its *r*^2^ imputation quality was 0.86.
*TMEM252*: The rs35179438 A allele at the *TMEM252* locus on chromosome 9 was associated with decreased *SHDB* gene expression (*P* = 2.7 × 10^−8^) (Table 4). None of the other studied gene expressions were associated with rs35179438.

**Table 4:** Association of *TMEM252* rs35179438 with human macrophage expression of 28 OXPHOS genes

Gene	Probes	Chr	Probe_Start	Probe_End	β*^a^*	SEM	*P* Value
M19 OXPHOS genes							
SDHB	*ILMN_1667257*	1	17,476,541	17,476,590	−0.053	0.0094	2.66 × 10^−8^
NDUFB3	*ILMN_2119945*	2	201,943,702	201,944,702	−0.034	0.0120	5.24 × 10^−3^
COX17	*ILMN_2187718*	3	119,396,160	119,396,209	−0.031	0.0139	0.0286
ATP5I	*ILMN_1772506*	4	678,058	678,107	−0.020	0.0090	0.0244
UQCRQ	*ILMN_1666471*	5	132,174,747	132,174,796	−0.045	0.0108	3.55 × 10^−5^
COX7A2	*ILMN_1701293*	6	75,950,943	75,950,943	−0.028	0.0104	7.53 × 10^−3^
ATP5J2	*ILMN_2307883*	7	99,217,929	99,217,978	−0.035	0.0112	1.58 × 10^−3^
NDUFB2	*ILMN_2117330*	7	140,402,713	140,402,762	−0.039	0.0122	1.44 × 10^−3^
COX6C	*ILMN_1654151*	8	100,904,152	100,904,201	−0.024	0.0083	4.07 × 10^−3^
COX8A	*ILMN_1809495*	11	63,742,263	63,743,263	−0.042	0.0108	1.16 × 10^−4^
NDUFA9	*ILMN_1760741*	12	4,796,151	4,796,200	−0.049	0.0124	1.04 × 10^−4^
ATP5G2	*ILMN_1660577*	12	54,063,071	54,063,120	−0.006	0.0133	0.675
COX6A1	*ILMN_1783636*	12	120,876,242	120,876,291	−0.018	0.0118	0.119
NDUFA11	*ILMN_2175712*	19	5,945,952	5,946,001	−0.028	0.0115	0.0142
NDUFB7	*ILMN_1813604*	19	14,816,068	14,817,068	−0.020	0.0114	0.0764
COX6B1	*ILMN_2154671*	19	36,139,232	36,139,281	−0.028	0.0094	0.0355
ATP5J	*ILMN_2348093*	21	28,180,168	28,180,217	−0.041	0.0109	1.63 × 10^−4^
NDUFA1	*ILMN_1784286*	X	119,005,887	119,005,936	−0.034	0.0099	6.42 × 10^−4^
M15 OXPHOS genes							
ATP5F1	*ILMN_1721989*	1	112,003,559	112,003,608	−0.022	0.0093	0.0195
PPA2	*ILMN_1687785*	4	106,292,029	106,293,029	−0.023	0.0148	0.123
NDUFC1	*ILMN_1733603*	4	140,216,254	140,217,254	0.007	0.0126	0.559
NDUFA4	*ILMN_1751258*	7	11,006,668	11,006,717	−0.007	0.0123	0.559
ATP5C1	*ILMN_1701269*	10	7,801,069	7,801,118	−0.032	0.0133	0.0157
SDHD	*ILMN_1698487*	11	111,966,144	111,966,193	−0.016	0.0145	0.262
ATP5L	*ILMN_2079285*	11	118,280,301	118,280,350	−0.013	0.0160	0.426
NDUFAB1	*ILMN_2179018*	16	23,684,934	23,684,983	−0.026	0.0138	0.0561
ATP5H	*ILMN_1666372*	17	75,524,607	75,524,656	−0.034	0.0097	5.44 × 10^−4^
NDUFV2	*ILMN_2086417*	18	9,126,871	9,127,871	−0.022	0.0125	0.0754

a Effect of the minor rs35179438 TA allele on gene expression. Its allele frequency was 0.25 and its *r*^2^ imputation quality was 0.79.
*PARK2*: The minor A allele of rs192804963 SNP, located in the *PARK2* gene, was significantly (*P* = 4.27 × 10^−8^) associated with increased *COX6C* expression and also demonstrated suggestive evidence for association (*P* < 10^−5^) with the expression of several other OXPHOS genes (Table 5). For 15 of the 28 OXPHOS gene expressions, the *PARK2* rs192804963 association *P*-value was < 0.01 (Table 5), a proportion (∼53%) was significantly higher (*P* = 5.2 × 10^−4^) than the corresponding proportion (23%, 1833 of 7862) observed in the remaining 7862 expressions. The rs192804963 effect on OXPHOS gene expressions was nearly codominant (Figure S3). We performed an eQTL analysis to identify other genes that could be under the genetic influence of the *PARK2* rs192804963 in human macrophages. Expression of four additional macrophage genes was significantly influenced by rs192804963 (*P* < 5 × 10^−8^), including *PRPSAP1* (*P* = 1.6 × 10^−8^), *PPME1* (*P* = 1.9 × 10^−8^), *CAMK2G* (*P* = 2.8 × 10^−8^), and *PTPN6* (*P* = 2.9 × 10^−8^). Of note, these four genes whose expression were modestly negatively correlated with those of the OXPHOS genes were not assigned to the preserved modules.

**Table 5:** Association of PARK2 rs192804963 with human macrophage expression of 28 OXPHOS genes

Gene	Probes	Chr	Probe_Start	Probe_End	β*^a^*	SEM	*P* Value
M19 OXPHOS genes							
SDHB	*ILMN_1667257*	1	17,476,541	17,476,590	0.027	0.0116	0.021
NDUFB3	*ILMN_2119945*	2	201,943,702	201,944,702	0.072	0.0143	5.22 × 10^−7^
COX17	*ILMN_2187718*	3	119,396,160	119,396,209	0.064	0.0167	1.36 × 10^−4^
ATP5I	*ILMN_1772506*	4	678,058	678,107	0.042	0.0107	9.34 × 10^−5^
UQCRQ	*ILMN_1666471*	5	132,174,747	132,174,796	0.035	0.0131	8.31 × 10^−3^
COX7A2	*ILMN_1701293*	6	75,950,943	75,950,943	0.055	0.0123	8.29 × 10^−6^
ATP5J2	*ILMN_2307883*	7	99,217,929	99,217,978	0.061	0.0133	5.13 × 10^−6^
NDUFB2	*ILMN_2117330*	7	140,402,713	140,402,762	0.035	0.0147	9.62 × 10^−3^
COX6C	*ILMN_1654151*	8	100,904,152	100,904,201	0.055	0.0098	4.27 × 10^−8^
COX8A	*ILMN_1809495*	11	63,742,263	63,743,263	0.056	0.0128	1.74 × 10^−5^
NDUFA9	*ILMN_1760741*	12	4,796,151	4,796,200	0.058	0.0152	1.08 × 10^−4^
ATP5G2	*ILMN_1660577*	12	54,063,071	54,063,120	0.024	0.0159	0.128
COX6A1	*ILMN_1783636*	12	120,876,242	120,876,291	0.071	0.0139	3.81 × 10^−7^
NDUFA11	*ILMN_2175712*	19	5,945,952	5,946,001	0.027	0.0139	0.055
NDUFB7	*ILMN_1813604*	19	14,816,068	14,817,068	0.026	0.0137	0.055
COX6B1	*ILMN_2154671*	19	36,139,232	36,139,281	0.022	0.0114	0.053
ATP5J	*ILMN_2348093*	21	28,180,168	28,180,217	0.023	0.0132	0.086
NDUFA1	*ILMN_1784286*	X	119,005,887	119,005,936	0.031	0.0119	0.010
M15 OXPHOS genes							
ATP5F1	*ILMN_1721989*	1	112,003,559	112,003,608	0.033	0.0112	3.32 × 10^−3^
PPA2	*ILMN_1687785*	4	106,292,029	106,293,029	0.000	0.0178	0.967
NDUFC1	*ILMN_1733603*	4	140,216,254	140,217,254	0.026	0.0152	0.085
NDUFA4	*ILMN_1751258*	7	11,006,668	11,006,717	0.051	0.0146	4.65 × 10^−4^
ATP5C1	*ILMN_1701269*	10	7,801,069	7,801,118	0.028	0.0160	0.079
SDHD	*ILMN_1698487*	11	111,966,144	111,966,193	0.027	0.0174	0.122
ATP5L	*ILMN_2079285*	11	118,280,301	118,280,350	0.009	0.0192	0.655
NDUFAB1	*ILMN_2179018*	16	23,684,934	23,684,983	0.052	0.0165	1.86 × 10^−3^
ATP5H	*ILMN_1666372*	17	75,524,607	75,524,656	0.020	0.0118	0.083
NDUFV2	*ILMN_2086417*	18	9,126,871	9,127,871	0.044	0.0150	3.52 × 10^−3^

a Effect of the minor rs192804963 A allele on gene expression. Its allele frequency was 0.21 and its *r*^2^ imputation quality was 0.66.

PARK2 gene expression in humans was tagged by two probes (ILMN_2395692 and ILMN_1714511) available on our array. However, none of them satisfied our QC criteria for detection *P*-values, the associated detection *P*-values being > 0.20 for > 95% of the samples. As a consequence, we were not able to test whether rs192804963 associates with *PARK2* macrophage expression in our study.

The *PARK2* rs192804963 is intronic and common, with an MAF of ∼0.20, and was inferred with a correct imputation quality of 0.66. According to public databases, it is in complete linkage disequilibrium (LD) (D′ = +1) with many other 3′ *PARK2* SNPs, including the genotyped rs75203550. The MAFs of the rs192804963 and rs75203550 slightly differed (0.21 *vs.* 0.13) in CTS, leading to a moderate pairwise LD *r*^2^ of ∼0.55. Nevertheless, the rs75203550 demonstrated a pattern of association with macrophage OXPHOS gene expressions similar to that observed with rs192804963 (Table S7). In addition, after adjusting for the effect of the genotyped rs75203550, the associations of rs192804963 with most OXPHOS gene expressions were no longer significant (Table S8). We were unable to test whether the PARK2 *trans* effect observed in human macrophages was also present in mice macrophages because the mouse study had very low power to assess this effect reliably.

**Figure 3:**
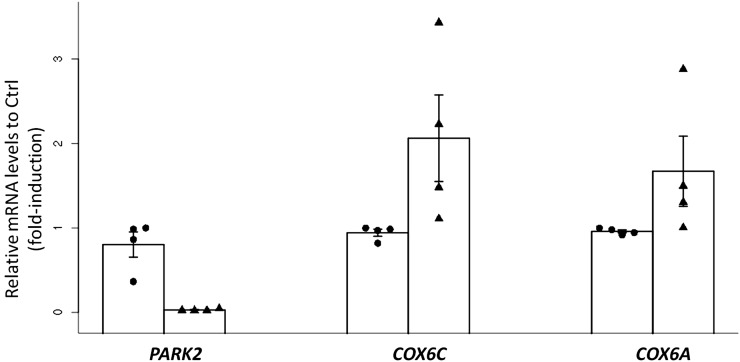
Increased *COX6A* and *COX6C* expression in *PARK2* KD human macrophages. Relative quantification of mRNA levels in human THP-1 macrophages transfected with control siRNA (circle) or siRNA targeting human PARK2 (triangle). The height of the open rectangle represents the mean (± SEM) over four independent experiments.

However, to follow-up on these epidemiological observations, we conducted preliminary experimental investigations to assess whether *PARK2* gene expression could associate *in vitro* with OXPHOS gene expressions in human THP-1 macrophages (see *Materials and Methods*). For this experimental work, we focused on *COX6C* and *COX6A* genes, the two OXPHOS genes whose expressions were the most significantly associated with rs192804963 (Table 5). As illustrated in Figure 3, KD PARK2 expression was accompanied with significant (*P* = 0.02) increase in *COX6C* and *COX6A* THP-1 expressions.

## Discussion

To our knowledge, this work is the first to propose a comprehensive approach investigating the genetic architecture of gene coexpression networks observed in human macrophages that are also preserved in mice. We provide strong evidence that genetic variability at *PARK2* gene influences the macrophage expression of several OXPHOS genes that are candidates for mitochondrial dysfunction, a biological pathway associated with several human diseases, such as neurological disorders.

Preservation analysis identified six gene coexpression modules in humans that were conserved in mouse transcriptome macrophage data. Four of these modules were significantly enriched into genes belonging to known biological pathways, such as ribosomal-associated proteins, proteasome-related gene, and oxidative phosphorylation. The OXPHOS pathway was of particular interest as several OXPHOS genes were annotated as susceptibility disease genes, in particular for diabetes and Alzheimer, Huntington, and Parkinson diseases (Table 2). These results are consistent with recent works reporting that oxidation phosphorylation could represent a key mechanism related to mitochondrial dysfunction pathway that could explain the association between type 2 diabetes and neurological disorders (Gibson 2005; Khan *et al.* 2014; Hao *et al.* 2015). Oxidative phosphorylation is an important component of mitochondrial function, and the later has previously been shown to be conserved between mouse and human brain transcriptome (Miller *et al.* 2010). In that sense, our results partially extend to macrophage some findings previously observed in brain. However, the preservation of the OXPHOS pathway between mouse and human does not appear to be ubiquitous as this pathway was not identified among the most commonly coexpressed genes in an extensive comparison across 30 different tissues (Monaco *et al.* 2015).

Because of the reported possible links between OXPHOS and human diseases, we further focused our genetic investigations on the OXPHOS genes and observed strong evidence of *trans*-association of *PARK2* rs192804963 with most macrophage OXPHOS gene expression. *PARK2* gene codes for Parkin, an E3 ubiquitin-protein ligase with rare missense mutations causing early onset Parkinson disease (Kitada *et al.* 1998). Several experimental works have shown that Parkin plays an important role in mitochondrial dysfunction by participating in mitochondria autophagic degradation (mitophagy) (Gehrke *et al.* 2015; Geisler *et al.* 2010; Narendra *et al.* 2010). The mode of action of Parkin in mitophagy is known to involve several partners, such as HDAC6, MFN1, MFN2, PINK1, SQSTM1, and VDAC1 (Narendra *et al.* 2010; Geisler *et al.* 2010; Lee *et al.* 2010; Chan *et al.* 2011; Gehrke *et al.* 2015). Interestingly, these genes were all expressed in our macrophage data but their expression was not associated with *PARK2* rs192804963 (all *P* > 0.05). Conversely, the strong associations of *PARK2* rs192804963 observed with most OXPHOS gene expressions open new perspectives into the downstream functions of Parkin. OXPHOS is known to associate with mitochondrial dysfunction (Breuer *et al.* 2013) but the precise mechanisms and the involved partners are not well understood. A recent experimental study (Gehrke *et al.* 2015) showed that Parkin participates in mRNA degradation of OXPHOS genes in HEK cells. Our results obtained from a large-scale epidemiological study, as well as those derived from experimental works that demonstrated PARK2 downregulation was associated with increased OXPHOS gene expression in human macrophages, are in line with this hypothesis. Our study additionally raises the hypothesis that the Parkin-dependent mRNA regulation of OXPHOS genes could be genetically determined. Altogether, these observations provide strong support for a role of Parkin in the regulation of genes participating in the OXPHOS biological system, and that this regulation is partially dependent on the genetic variability of the PARK2 locus. Due to the emerging links between OXPHOS, neurological disorders (*e.g.*, Alzheimer and Parkinson), and diabetes (Lima *et al.* 2014; De Felice and Ferreira 2014; Santiago and Potashkin 2014), it is tempting to hypothesize that the identified *PARK2* polymorphisms could impact the risk of such human diseases. Unfortunately, the PARK2 variants discussed in this work were not available in the IGAPS, IPDGC, or DIAGRAM public depository for GWAS results in Alzheimer, Parkinson, and type 2 diabetes diseases, respectively. This is likely due to the fact that these results were not obtained through 1000 Genomes imputation. Conversely, *PARK2* is a susceptibility gene for leprosy (Mira *et al.* 2004), and de Léséleuc *et al.* (2013) have reported that polymorphisms mapping to the *PARK2* promoter region could also exert some regulator effect in *trans* on the secretion of inflammatory cytokines. As the *PARK2* SNP identified in our work do not show any LD with PARK2 promoter polymorphisms, it would be tempting to hypothesize that Parkin could have a pleiotropic influence on several biological mechanisms through different genetic regulations. Several investigations, including a fine mapping of the whole *PARK2* locus, would be mandatory to assess this hypothesis.

Several limitations must be acknowledged. First, macrophages are heterogeneous cells that may have different regulations and functions according to tissue specificity (Pollard 2009). In our study, mouse macrophages were primary peritoneal macrophage cells, while in humans, macrophages were generated from monocytes by M-CSF stimulation. RNA preparation, microarray hybridization, and expression data preprocessing were performed in different laboratories and followed different bioinformatics workflows. Nevertheless, such discrepancies may be considered as strengths as they introduced positive preferential bias in favor of genes ubiquitously expressed in the most common macrophage cell types. Second, our strategy for preservation analysis of gene expression modules between mouse and human was based on first identifying modules in human and then assessing whether these were preserved in mouse. Several parameters had to be fixed at different steps of the analysis workflow, such as the minimum size of the modules and the β power used in transforming the correlation matrix to an adjacent matrix satisfying scale-free topology criteria. We performed sensitivity analyses by modifying these parameters and observed similar findings (data not shown). Third, we report here the results of the preservation of human modules in the mouse dataset. We also conducted the module identification in mouse (despite the much smaller sample size) and assessed their preservation in human. Similar findings were observed; for example, modules enriched for ribosome genes (FDR of ∼10^−8^) and oxidative phosphorylation (FDR of ∼10^−5^) were identified as preserved. Fourth, our preservation analysis and genetic investigations relied on the use of the ME approach. This strategy may not fully detect the preserved modules and the genetic variations underlying their expression variability, as the percentage of module expression variability explained by the ME was rather moderate. By design (Charchar *et al.* 2012; Garnier *et al.* 2013), the CTS dataset was composed of individuals affected with coronary artery disease and healthy individuals. Even though we cannot exclude that this may have introduced additional heterogeneity in the study sample, it is important to emphasize that the *trans* effect observed at *PARK2* rs192840963 is present both in healthy and diseased individuals (Table S9). Finally, our results were mainly derived from a comprehensive epidemiological investigation of large-scale and well-powered genomic/transcriptomic resources. It was not possible to replicate the statistical associations/correlations we observed in macrophages, as there are no other human epidemiological resources available that are similar to CTS resources. This is an important point, especially for the *trans* association observed at the lead *PARK2* SNP that was imputed. Even though its imputation quality was correct, validation of the observed association on genotyped SNP data could be valuable. However, it was not possible to test it in the present study as we did not have easy access to DNA samples of the studied individuals. Further experimental works, including *PARK2* KD in mice, are mandatory to support our findings.

In conclusion, this study provides new arguments supporting the role of Parkin as a key regulator of oxidative phosphorylation in macrophages, and suggests that this mechanism could be partially genetically determined in humans.

## Supplementary Material

Supplemental Material
