# Supplementary material for: Preservation Analysis of Macrophage Gene Coexpression Between Human and Mouse Identifies PARK2 as a Genetically Controlled Master Regulator of Oxidative Phosphorylation in Humans
Source: G3 (Bethesda). 2016 Aug 24;6(10):3361–71. doi: 10.1534/g3.116.033894 (PMC5068955; doi:10.1534/g3.116.033894)
Supplement: Supplemental Material [file supp_6_10_3361__index.html]

Preservation Analysis of Macrophage Gene Coexpression Between Human and Mouse Identifies PARK2 as a Genetically Controlled Master Regulator of Oxidative Phosphorylation in Humans — Supplemental Material 

# Preservation Analysis of Macrophage Gene Coexpression Between Human and Mouse Identifies PARK2 as a Genetically Controlled Master Regulator of Oxidative Phosphorylation in Humans

## Supplemental Material for Codoni *et al.*, 2016

**Files in this Data Supplement:**

- Table S1 - Primers used for quantification of *PARK2*, *COX6A* and *COX6C* mRNA by QPCR. (.pdf, 169 KB)
- Table S7 - Association of PARK2 rs75203550 with human macrophage expression of 28 OXPHOS genes. (.pdf, 283 KB)
- Table S8 - Association of PARK2 rs192804963 with human macrophage expression of 28 OXPHOS genes after adjusting for the effect of PARK2 rs75203550. (.pdf, 284 KB)
- Figure S1 - Scatter plot of the kME values observed in human and mouse for the genes assigned to the M19 gene module. (.tif, 110 KB)
- Figure S2 - Module preservation analysis based on composite preservation statistics. (.tif, 416 KB)
- Figure S3 - Association of PARK2 rs192804963 on some human macrophage OXPHOS gene expression. (.tif, 78 KB)
- Table S2 - Gene composition of the six most preserved modules. (.xlsx, 17 KB)
- Table S3 - Main results of the trans eQTL analyses observed in Proteasome genes. (.xlsx, 10 KB)
- Table S4 - Main results of the cis eQTL analyses observed in Ribosome genes. (.xlsx, 72 KB)
- Table S5 - Main results of the trans eQTL analyses observed in Ribosome genes. (.xlsx, 10 KB)
- Table S6 - Main results of the cis eQTL analyses observed in OXPHOS genes. (.xlsx, 20 KB)
- Table S9 - Association of PARK2 rs192804963 with human macrophage expression of 28 OXPHOS genes separately in healthy and CAD individuals. (.xlsx, 13 KB)
