# Supplementary material for: Preservation Analysis of Macrophage Gene Coexpression Between Human and Mouse Identifies PARK2 as a Genetically Controlled Master Regulator of Oxidative Phosphorylation in Humans
Source: G3 (Bethesda). 2016 Aug 24;6(10):3361–71. doi: 10.1534/g3.116.033894 (PMC5068955; doi:10.1534/g3.116.033894)
Supplement: Supplemental Material [file supp_g3.116.033894_TableS1.pdf]

Table S1. Primers used for quantification of *PARK2*, *COX6A* and *COX6C* mRNA by QPCR.

	Forward Primer	Reverse Primer
**Human PARK2**	5'-CCACACTGCCCTGGGACTA-3'	5'-GACGGGGCTCCTGACG-3'
**Human COX6A1**	5'-CTCGCATGTGGAAGACTCTC-3'	5'-AACGGCTTGGTCCTGATG-3'
**Human COX6C**	5'-CTTTGTATAAGTTTCGTGTGG-3'	5'-ATTCATGTGTCATAGTTCAGG-3'
**Human delta-aminolvulinate synthase**	5'-CTGCAAGCAAATGCCCTTTC-3'	5'-CCCTCCATCGGTTTTCACAC-3'
**Human hypoxanthine phosphoribosyltransférase 1**	5'-TGACACTGGCAAAACAATGC-3'	5'-AACACTTCGTGGGGTCCTTT-3'
**Human alpha-tubuline**	5'-GATGCTGCCAATAACTATGCCCGAG-3'	5'-GAAAACCAAGAAGCCCTGAAGACGG-3'
