# Supplementary material for: Preservation Analysis of Macrophage Gene Coexpression Between Human and Mouse Identifies PARK2 as a Genetically Controlled Master Regulator of Oxidative Phosphorylation in Humans
Source: G3 (Bethesda). 2016 Aug 24;6(10):3361–71. doi: 10.1534/g3.116.033894 (PMC5068955; doi:10.1534/g3.116.033894)
Supplement: Supplemental Material [file supp_g3.116.033894_TableS7.pdf]

Table S7. Association of PARK2 rs75203550 with human macrophage expression of 28 OXPHOS genes

Gene	Probes	Chr	Beta*	Se	Pvalue
Light-yellow OxPho genes					
SDHB	*ILMN_1667257*	1	0.022	0.0124	0.075
NDUFB3	*ILMN_2119945*	2	0.063	0.0154	$4.31\ 10^{-5}$
COX17	*ILMN_2187718*	3	0.060	0.0178	$8.35\ 10^{-4}$
ATP5I	*ILMN_1772506*	4	0.026	0.0115	0.026
UQCRQ	*ILMN_1666471*	5	0.034	0.0139	0.014
COX7A2	*ILMN_1701293*	6	0.054	0.0132	$5.85\ 10^{-5}$
ATP5J2	*ILMN_2307883*	7	0.050	0.0143	$4.83\ 10^{-4}$
NDUFB2	*ILMN_2117330*	7	0.038	0.0158	0.0174
COX6C	*ILMN_1654151*	8	0.053	0.0105	$4.04\ 10^{-7}$
COX8A	*ILMN_1809495*	11	0.052	0.0138	$1.88\ 10^{-4}$
NDUFA9	*ILMN_1760741*	12	0.054	0.0160	$8.93\ 10^{-4}$
ATP5G2	*ILMN_1660577*	12	0.028	0.0171	0.105
COX6A1	*ILMN_1783636*	12	0.072	0.0148	$1.82\ 10^{-6}$
NDUFA11	*ILMN_2175712*	19	0.028	0.0148	0.052
NDUFB7	*ILMN_1813604*	19	0.022	0.0146	0.129
COX6B1	*ILMN_2154671*	19	0.018	0.0121	0.138
ATP5J	*ILMN_2348093*	21	0.025	0.0141	0.076
NDUFA1	*ILMN_1784286*	X	0.031	0.0128	0.017
Midnight-blue OxPho genes					
ATP5F1	*ILMN_1721989*	1	0.037	0.0119	$1.86\ 10^{-3}$
PPA2	*ILMN_1687785*	4	-0.0182	0.0190	0.338
NDUFC1	*ILMN_1733603*	4	0.019	0.0162	0.239
NDUFA4	*ILMN_1751258*	7	0.052	0.0156	$8.92\ 10^{-4}$
ATP5C1	*ILMN_1701269*	10	0.018	0.0171	0.290
SDHD	*ILMN_1698487*	11	0.014	0.0186	0.443
ATP5L	*ILMN_2079285*	11	0.008	0.0206	0.678
NDUFAB1	*ILMN_2179018*	16	0.040	0.0178	0.025
ATP5H	*ILMN_1666372*	17	0.015	0.0126	0.244
NDUFV2	*ILMN_2086417*	18	0.044	0.0161	$6.68\ 10^{-3}$

* Effect of the minor rs75203550-T allele on gene expression. Its allele frequency was 0.13.
