# Supplementary material for: Preservation Analysis of Macrophage Gene Coexpression Between Human and Mouse Identifies PARK2 as a Genetically Controlled Master Regulator of Oxidative Phosphorylation in Humans
Source: G3 (Bethesda). 2016 Aug 24;6(10):3361–71. doi: 10.1534/g3.116.033894 (PMC5068955; doi:10.1534/g3.116.033894)
Supplement: Supplemental Material [file supp_g3.116.033894_TableS8.pdf]

Table S8. Association of PARK2 rs192804963 with human macrophage expression of 28 OXPHOS genes after adjusting for the effect of PARK2 rs75203550

Gene	Probes	Chr	Beta*	Se	Pvalue
Light-yellow OxPho genes					
SDHB	*ILMN_1667257*	1	0.027	0.0185	0.142
NDUFB3	*ILMN_2119945*	2	0.065	0.0227	$4.23\ 10^{-3}$
COX17	*ILMN_2187718*	3	0.049	0.0265	0.063
ATP5I	*ILMN_1772506*	4	0.060	0.017	$4.54\ 10^{-4}$
UQCRQ	*ILMN_1666471*	5	0.024	0.021	0.254
COX7A2	*ILMN_1701293*	6	0.042	0.019	0.034
ATP5J2	*ILMN_2307883*	7	0.062	0.021	$3.36\ 10^{-3}$
NDUFB2	*ILMN_2117330*	7	0.028	0.023	0.226
COX6C	*ILMN_1654151*	8	0.037	0.015	0.016
COX8A	*ILMN_1809495*	11	0.043	0.020	0.035
NDUFA9	*ILMN_1760741*	12	0.049	0.024	0.039
ATP5G2	*ILMN_1660577*	12	0.010	0.025	0.689
COX6A1	*ILMN_1783636*	12	0.048	0.022	0.029
NDUFA11	*ILMN_2175712*	19	0.014	0.022	0.524
NDUFB7	*ILMN_1813604*	19	0.026	0.219	0.222
COX6B1	*ILMN_2154671*	19	0.022	0.018	0.226
ATP5J	*ILMN_2348093*	21	0.009	0.021	0.655
NDUFA1	*ILMN_1784286*	X	0.021	0.019	0.269
Midnight-blue OxPho genes					
ATP5F1	*ILMN_1721989*	1	0.014	0.018	0.425
PPA2	*ILMN_1687785*	4	0.032	0.028	0.251
NDUFC1	*ILMN_1733603*	4	0.030	0.024	0.219
NDUFA4	*ILMN_1751258*	7	0.032	0.023	0.161
ATP5C1	*ILMN_1701269*	10	0.037	0.025	0.143
SDHD	*ILMN_1698487*	11	0.041	0.027	0.134
ATP5L	*ILMN_2079285*	11	0.038	0.031	0.215
NDUFAB1	*ILMN_2179018*	16	0.057	0.026	0.030
ATP5H	*ILMN_1666372*	17	0.025	0.019	0.191
NDUFV2	*ILMN_2086417*	18	0.034	0.024	0.162

* Effect of the minor rs192804963-A allele on gene expression. Its allele frequency was 0.21 and its r^2 imputation quality 0.66.
