# Supplementary figures and images for: Preservation Analysis of Macrophage Gene Coexpression Between Human and Mouse Identifies PARK2 as a Genetically Controlled Master Regulator of Oxidative Phosphorylation in Humans
Source: G3 (Bethesda). 2016 Aug 24;6(10):3361–71. doi: 10.1534/g3.116.033894 (PMC5068955; doi:10.1534/g3.116.033894)

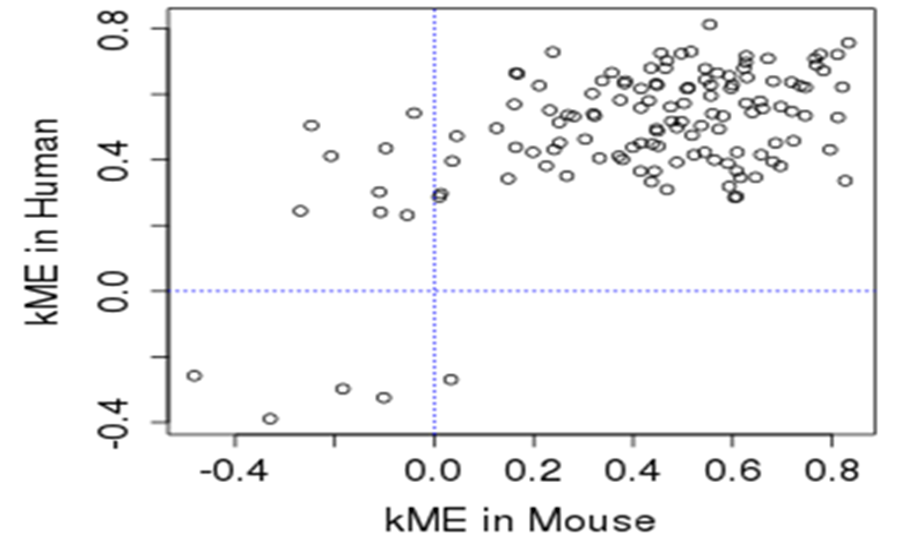

Supplement: Supplemental Material [file supp_g3.116.033894_FigureS1.tif]

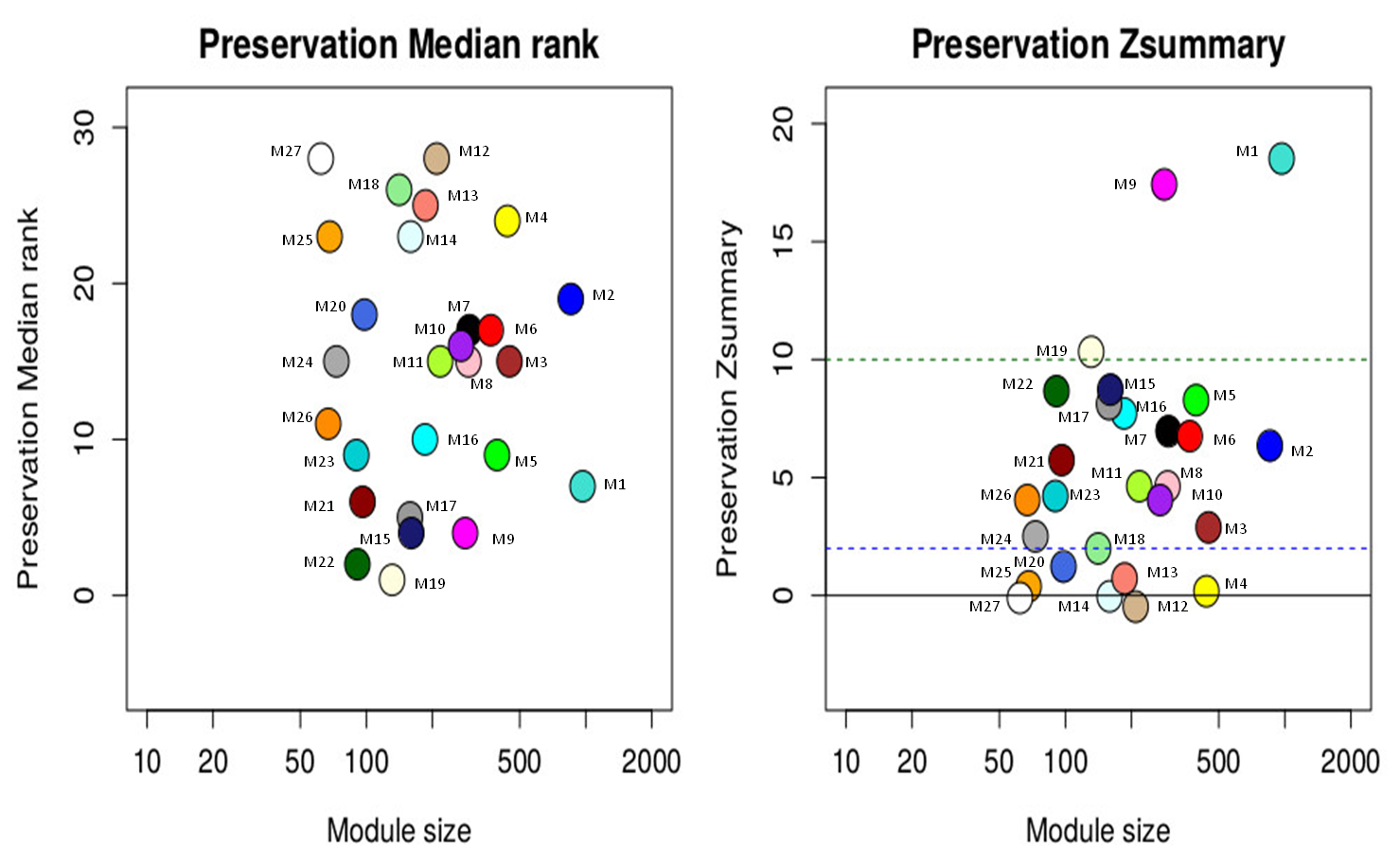

Supplement: Supplemental Material [file supp_g3.116.033894_FigureS2.tif]

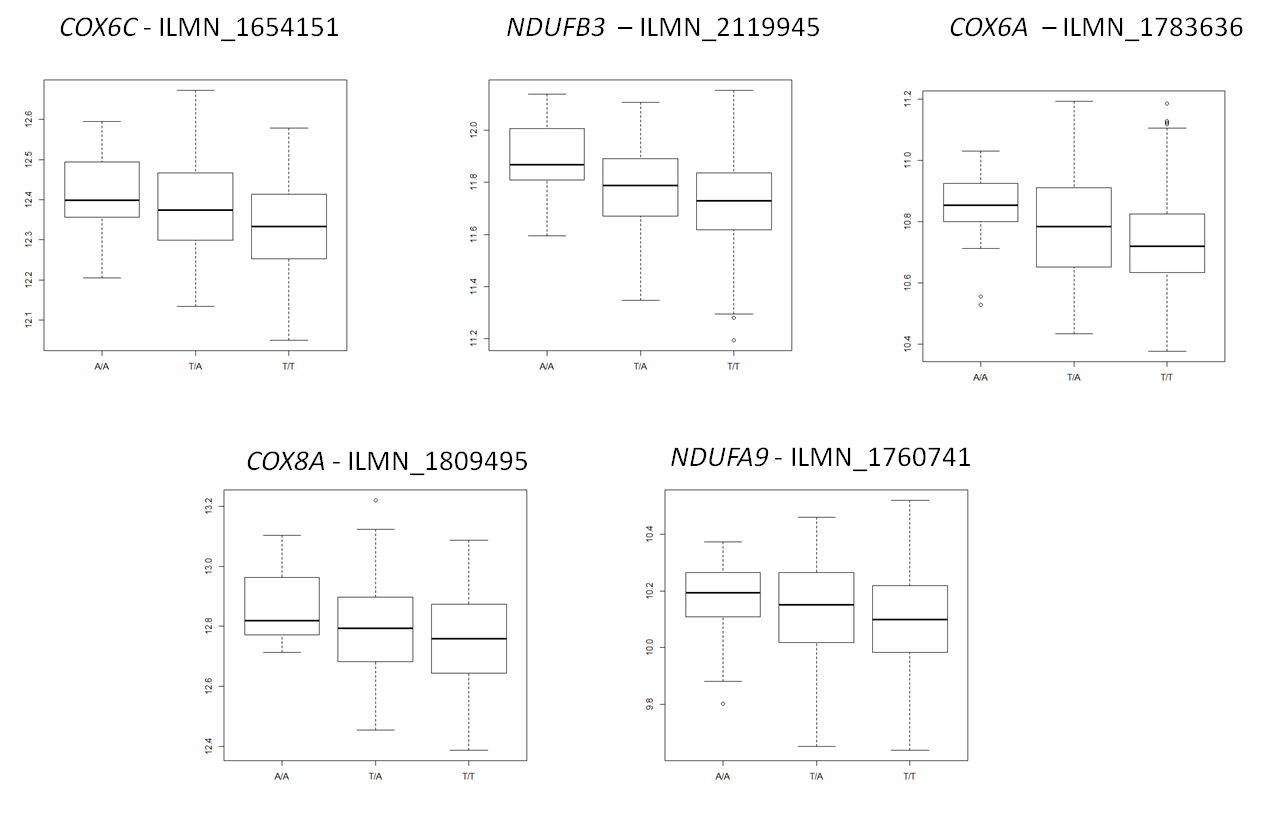

Supplement: Supplemental Material [file supp_g3.116.033894_FigureS3.tif]
